# Ko Kuei Chen: a pioneer of modern pharmacological research in China

**DOI:** 10.1093/procel/pwac049

**Published:** 2022-11-04

**Authors:** Huan Liu, Zhaoqi Liu, Xue Gong, Hao Cheng

**Affiliations:** University of Science and Technology of China, Hefei 230026, China; State Key Laboratory of Virology, Wuhan 430072, China; University of Science and Technology of China, Hefei 230026, China; University of Science and Technology of China, Hefei 230026, China; Institute of Microbiology, Chinese Academy of Sciences, Beijing 100101, China

Ko Kuei Chen (陈克恢, 1898–1988) (Fig. 1) was a pharmacologist who ceaselessly strove for medical research all his life. He was a pioneer of modern pharmacological research in Chinese medicine (Qian, 2014).

**Figure 1.**
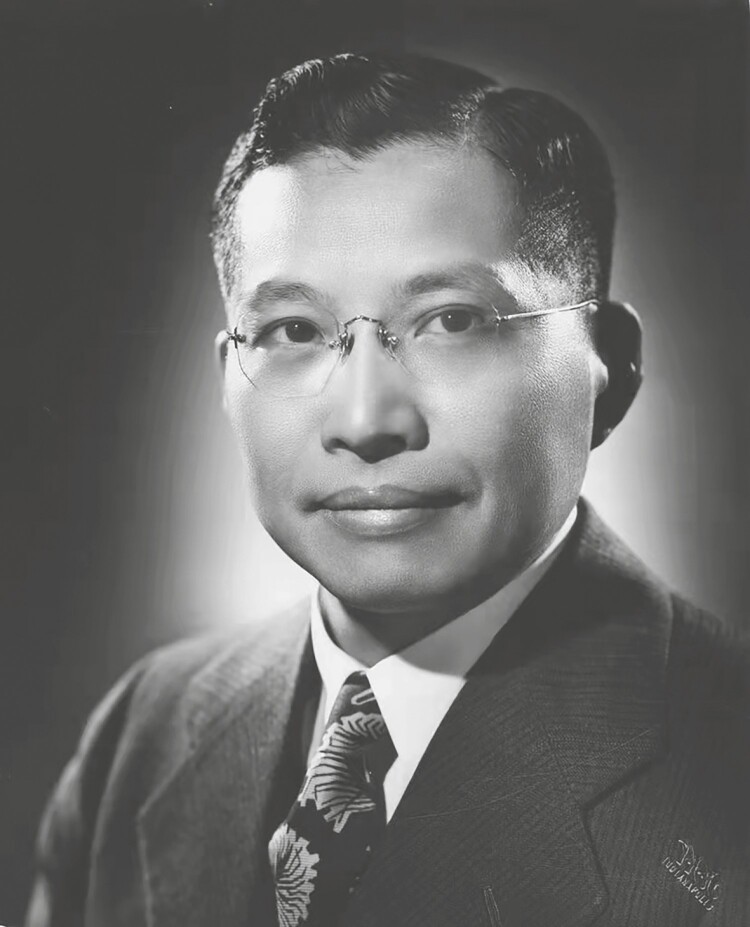
Ko Kuei Chen: a pioneer of modern pharmacology in Chinese medicine.

He systematically studied the pharmacological action of ephedrine and published the first paper on this topic in the world. He discovered that injecting sodium nitrite and sodium thiosulfate intravenously could effectively treat acute cyanide poisoning (Chen and Rose, 1952). He isolated cinobufagin and cinobufotoxin from Ch’an Su, the dried venom of the Chinese toad. His research in pharmacology enriched the treasure trove of medicine. Ko Kuei Chen was a pioneer of pharmacology in Chinese medicine, and his research had epoch-making significance in the field. His pharmacological research on ephedrine set a milestone in the world medicine history.

## Ko Kuei Chen’s education

**Figure 2.**
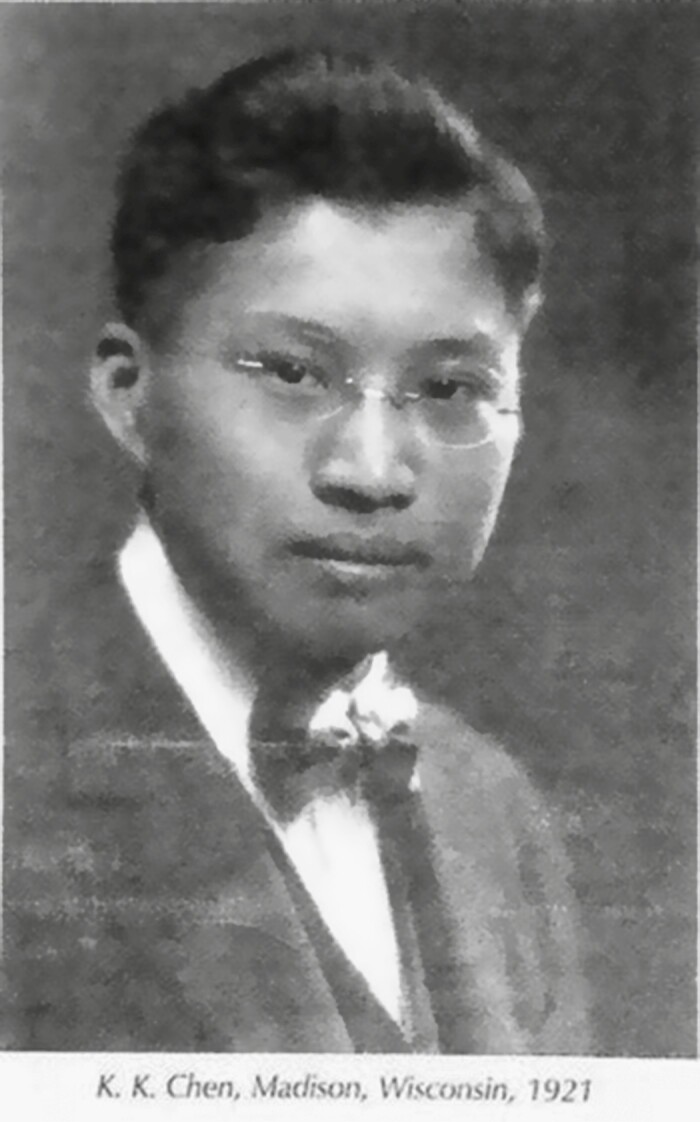
Ko Kuei Chen, Madison, Wisconsin, 1921.

When Ko Kuei Chen was young, his father died and he was raised by his uncle Shounan Zhou (周寿南), a Chinese physician. Due to his influence, Ko Kuei Chen became interested in Chinese herbal medicine at an early age. In 1916, he was admitted to “Tsing Hua College” the predecessor of Tsinghua University. In 1918, Ko Kuei Chen graduated from Tsinghua University (Ding, 2009). In 1920, Chen conducted research on cassia oil in the School of Pharmacy at University of Wisconsin-Madison, where he completed his thesis and received a bachelor of science degree (Chen, 1923) (Fig. 2).

In 1923, he received a Ph.D. in Physiology and Pathology Sciences at University of Wisconsin-Madison. From 1923 to 1925, Chen served as an assistant professor in the Department of Pharmacy at Peking Union Medical College. In 1927, he received his M.D. from Johns Hopkins University and was promoted to an associate professor in pharmacology. In 1929, he became the director of the research department of Eli Lilly and Company. From 1937 to 1968, Chen was a Professor of Pharmacology at Indiana University School of Medicine. In 1968, Chen retired from Indiana University (Qian, 2014).

## Pharmacological action of ephedrine

Ko Kuei Chen is best known for his research on ephedrine. The discovery of ephedrine’s pharmacological action was of epoch-making significance in the history of medicine. Ko Kuei Chen was the first to reveal the pharmacological action of ephedrine and apply it in clinical treatment.

**Figure 3.**
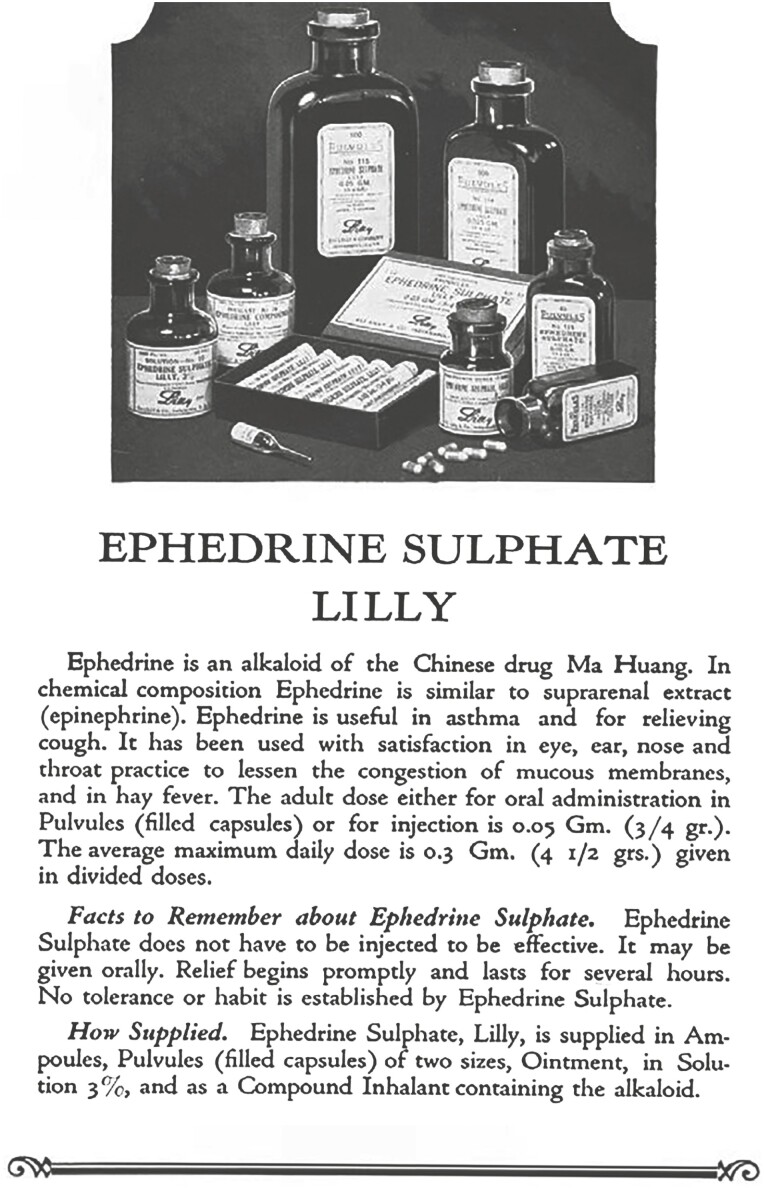
Introduction of ephedrine sulfate.

In 1923, when Ko Kuei Chen was at Peking Union Medical College, in addition to teaching, he wished to continue his research on Chinese herbal medicine, and received support from Prof. Carl Frederic Schmidt, the dean of the department. Ko Kuei Chen learned from his uncle Shounan Zhou that Ma Huang could relieve asthma. At his uncle’s suggestion, he chose Ma Huang among hundreds of commonly used Chinese medicinal herbs as the first research object. Ephedrine was successfully isolated from Ma Huang using the ammonia–chloroform method in just a few weeks (Chen and Schmidt, 1926). Previously, Nagai Nagayoshi had separated ephedrine from Ma Huang in 1887 and named it ephedrine, noting that it can cause pupil dilatation. Chen and Schmidt studied the pharmacological action of ephedrine in detail, and found that ephedrine could increase the carotid pressure for a long time, enhance cardiac contractions, constrict splenic and renal vessels, as well as mucous membrane and skin, and relax bronchial muscles (Qian, 2014). Ephedrine could stimulate the isolated uterus and central nervous system, and cause pupil dilatation after dripping it into eyes (Chen and Schmidt, 1926). These effects of ephedrine are qualitatively identical with those of epinephrine, while ephedrine can be taken orally, with longer efficacy duration and lower toxicity. In 1924, Chen and Schmidt made a preliminary report at the Beijing branch venue of the Society for Experimental Biology and Medicine. In the same year, after systematic experiments, they wrote their research paper “The action of ephedrine, the active principle of Chinese drug Ma Huang,” and published it in the magazine “*Journal of Pharmacology and Experimental Therapeutics*,” which was the first paper on the pharmacological action of ephedrine in the world eyes (Chen and Schmidt, 1924). The physiological effects of ephedrine, the active principle of Ma Huang, are similar to epinephrine and last longer, primarily acting via the stimulation of the sympathetic nervous system. Since then, ephedrine has become an important sympathomimetic drug, which attracted international attention (Fig. 3). The study was a model for identifying lead compounds from natural products and optimizing them to develop new drugs. Ko Kuei Chen is reputed as a pioneer of modern pharmacological research in Chinese medicine.

## Technique for treating acute cyanide poisoning

In the early 1930s, Ko Kuei Chen and his colleagues discovered a technique for treating acute cyanide poisoning. On the basis of previous research, Chen and his research team found that the combination of sodium nitrite and sodium thiosulfate, consecutively injected intravenously, detoxifies cyanide to a marked degree (Chen and Rose, 1952). Furthermore, they also found that the combination of nitrite and thiosulfate has a synergistic effect that surpasses the sum of their individual values (Chen and Rose, 1952). At present, nitrite–thiosulfate therapy is still an effective detoxification method in the treatment of acute cyanide poisoning, based on the intravenous administration of sodium nitrite, followed by the administration of sodium thiosulfate.

## Research on Ch’an Su

Ko Kuei Chen spent much of his life studying Ch’an Su, the dried venom of Chinese toad. Since 1927 when he was in Abel’s Laboratory, he had shown great interest in Ch’an Su. Ko Kuei Chen was the first to have succeeded in obtaining epinephrine and the N-containing compound from Ch’an Su in crystalline form (Chen et al., 1931). Soon after, Ko Kuei Chen isolated the two pure compounds cinobufagin and cinobufotoxin from Ch’an Su, and found that these two components had a digitalis-like cardiotonic effect. Compared with digoxigenin, cinobufagin had a shorter duration of action and was ineffective when taken orally. In 1929, after Ko Kuei Chen came to Eli Lilly and Company, he continued his research on Ch’an Su for more than 40 years (Song, 1996). He studied the structure–activity relationship of more than 400 cardiac glycosides and steroids. Furthermore, he also found that animals other than toads were capable of storing glycosides of the cardenolide type from their plant foods (Chen, 1970). Ko Kuei Chen published a number of articles that have enriched the treasure trove of medicinal chemistry and provided valuable reference for the study of other drugs.

## Other pharmacological studies

In the 1940s, Ko Kuei Chen found that γ-dichroine extracted from the Chinese medicinal herb Ch’ang Shan (*Dichroa febrifuga*) is antimalarial, and its effect is 148 times stronger than that of quinine (Henderson et al., 1949). Since it causes vomiting, hepatic edema, and degeneration, γ-dichroine was not introduced into clinical practice. Shortly after World War II, Ko Kuei Chen obtained a sample of methadone from Germany and confirmed its analgesic effect (Chen, 1948). On this basis, he and his colleagues synthesized and developed propoxyphene in Eli Lilly and Company (Qian, 2014). Although its clinical effect is equal to or slightly worse than that of codeine, it is less addictive, and it exhibits synergism with aspirin.

## Master of international medicine

Ko Kuei Chen engaged in pharmacology for more than 50 years and published more than 350 papers and reviews. Due to his extensive and in-depth research interests, he made great contributions to the development of new drugs.

Ko Kuei Chen separated and extracted active ingredients from traditional Chinese medicinal materials and developed them into chemical drugs. Following this approach, Chinese medical scientists and chemists have carried out numerous studies and achieved major breakthroughs, such as the discovery of the new antihepatitis drugs bifendate and bicyclol, as well as the antimalarial drugs artemisinin and dihydroartemisinin, etc. Ko Kuei Chen’s academic thoughts have far-reaching significance for the research of Chinese herbal medicine as well as other natural and synthetic drugs. His scientific thoughts and research methods have walked broad road and greatly promoted pharmacological research in modern Chinese medicine.
